# Appetite in Palliative Cancer Patients and Its Association with Albumin, CRP and Quality of Life in Men and Women—Cross-Sectional Data from the Palliative D-Study

**DOI:** 10.3390/life12050671

**Published:** 2022-04-30

**Authors:** Charlotte Goodrose-Flores, Stephanie Bonn, Caritha Klasson, Maria Helde Frankling, Ylva Trolle Lagerros, Linda Björkhem-Bergman

**Affiliations:** 1 Clinical Epidemiology Division, Department of Medicine (Solna), Karolinska Institutet, 171 77 Solna, Sweden; stephanie.bonn@ki.se (S.B.); ylva.trolle@ki.se (Y.T.L.); 2 Division of Geriatrics, Department of Neurobiology, Care Sciences and Society (Huddinge), Karolinska Institutet, 171 77 Solna, Sweden; caritha.klasson@ki.se (C.K.); maria.helde.frankling@ki.se (M.H.F.); linda.bjorkhem-bergman@ki.se (L.B.-B.); 3 Theme Cancer, Karolinska University Hospital, 112 19 Stockholm, Sweden; 4 Center of Obesity, Academic Specialist Center, Stockholm Health Services, 112 19 Stockholm, Sweden

**Keywords:** cancer, palliative care, appetite, quality of life, sex differences, pain, fatigue, albumin

## Abstract

Albumin is an important biochemical marker in palliative cancer care, used for assessment of nutritional status, disease severity and prognosis. Our primary aim was to investigate sex differences in the association between appetite and albumin levels in palliative cancer patients. We also aimed to study associations between appetite and C-reactive protein (CRP), Quality of Life (QoL), pain and fatigue. In the Palliative D-cohort, consisting of 266 men and 264 women, we found a correlation between appetite and albumin; low appetite, measured with the Edmonton Symptom Assessment System, correlated significantly with low albumin in men: (r = −0.33, *p* < 0.001), but not in women (r = −0.03, *p* = 0.65). In a regression analysis adjusted for confounding factors, results were similar. Lower appetite was correlated with higher CRP in men (r = 0.27, *p* < 0.001), but not in women (r = 0.12, *p* = 0.05). Appetite was correlated with QoL, fatigue and pain in both men and women; those with a low appetite had a low QoL and high fatigue- and pain-scores (*p* < 0.001). In conclusion, our results indicated possible sex differences in the associations between appetite and albumin, and between appetite and CRP, in palliative care patients. Understanding these associations could provide additional value for clinical practice.

## 1. Introduction

The aim of palliative care is to provide relief from distressing symptoms to maintain a high quality of life (QoL) in patients facing a life-threatening illness. One distressing symptom that is often overlooked is loss of appetite. 

A low appetite is common among cancer patients. It is associated with malnutrition and has been shown to influence QoL [1,2]. Cancer cachexia occurs in 80 percent of patients diagnosed with advanced cancer [3]; it is commonly accompanied by low levels of albumin, and is associated with worse outcomes [4]. Loss of appetite and the subsequent reduction in energy intake [5] contributes to the progression of the three consecutive phases of cancer cachexia [6]. The initial stage, pre-cachexia, includes a 2–5% weight loss and metabolic changes such as insulin resistance and inflammation. The second stage, cancer cachexia, is diagnosed when weight loss exceeds 5% over the preceding 6 months, and in the third stage, refractory cachexia, there is a loss of skeletal muscle and adipose tissue along with a catabolic state, with symptoms exacerbating as the cancer progresses [3,6].

The pathophysiology of appetite regulation in cancer cachexia is complex [6]. The interrelations between appetite, food intake and weight loss are multifactorial and not clearly defined [5,7]. A contributing factor to decreased appetite is altered hypothalamic control of appetite and satiety [8]. Other factors include a deranged metabolism, involving the release of hormone-like cytokines, which separate cancer cachexia from simple malnutrition [6,9]. Further, symptoms of cancer-related eating difficulties, such as nausea, vomiting, dysphagia and dysgeusia, might be experienced by the patients as interrelated. However, the distinction of symptoms is important as they are targeted by different palliative treatments in the clinical setting [10].

In clinical practice, nutritional status in cancer patients is often assessed by monitoring albumin levels. Albumin is synthesized in the liver and is the most abundant protein in human serum [11,12,13]. In healthy adults, the reference range for albumin concentration is usually between 36 and 40 g/L [14]. Albumin concentration reaches a peak at approximately 20 years of age, and decreases thereafter by approximately 0.1 g/L per year of life [11,13]. The decline is more rapid in women, but from age 60 years the concentration is similar for men and women [13]. Decreased albumin levels are observed during episodes of inflammation and infections. Hypoalbuminemia is common in cancer patients [15,16], but it is not found in individuals with starvation-related malnutrition, as there is no inflammation present [17]. Therefore, the impact of albumin as a predictive measure to identify malnutrition is weak, due to its lack of specificity and its long half-life [11,14]. However, it is well established that hypoalbuminemia is a prognostic marker for increased mortality in cancer patients [18,19].

Elevated C-reactive protein (CRP) levels, indicating inflammation, infection, or progress of cancer, have also been associated with decreased appetite [20,21]. However, whether there are associations between appetite and albumin, or between appetite and CRP, that differ between men and women has not previously been studied in a palliative cancer population. Sex differences are gaining more attention within the field of oncology. They are considered a possible modulator of disease biology, as they impact areas such as cancer biology, immune system activity and body composition [21]. 

As mentioned above, maintaining QoL is the overall goal in palliative care. However, there is no consensus regarding the definition of QoL, nor is there any clear definition between QoL and similar terms, such as Health Related Quality of Life or Health Status [22]. The World Health Organization (WHO) states that QoL is “an individual’s perception of their position in life in the context of the culture and value systems in which they live and in relation to their goals, expectations, standards and concerns” [23]. Thus, the concept of QoL is subjective and tied to personal values, but is also affected by the value of community [24]. In palliative care, QoL is measured as the individual experience, and is usually self-assessed using a numeric scale [25,26]. A patient’s assessment of what comprises a good QoL, or a poor one, may vary substantially as the disease progresses [27]. 

Fatigue and pain are common distressing symptoms in palliative cancer patients which affect QoL. Fatigue is severe tiredness and weakness that is not improved by sleep or rest [28]. The etiology of cancer-related fatigue remains unknown, and is probably multi-factorial, although inflammation is thought to be a major contributing factor [28]. This is supported by the notion that fatigue is associated with increased CRP levels [20]. Fatigue in advanced cancer patients has also been associated with the presence of malnutrition [29]. However, there is still a knowledge gap regarding how fatigue and pain are associated with appetite, and whether there are differences between men and women. 

The primary aim of our study was to investigate differences between men and women in the association between self-assessed appetite and measured albumin levels in palliative cancer patients. The secondary aim was to study associations between appetite and CRP, QoL, pain and fatigue. 

## 2. Materials and Methods

### 2.1. Study Design and Population

In this cross-sectional study we performed a post hoc analysis of baseline data from the Palliative-D study [30]. In brief, the Palliative-D study was a double-blind, randomized, placebo-controlled, multi-center trial comprising patients with advanced cancer admitted to palliative care [30]. In total, 530 patients were recruited from three Advanced Medical Home Care units (ASIH) in Stockholm during 2017–2020: ASIH Stockholm Södra, ASIH Stockholm Norr and Stockholm’s Sjukhem ASIH. 

Inclusion criteria were men and women, ≥18 years old, diagnosed with advanced and/or metastatic cancer in the palliative phase, and with a life expectancy >3 months. More details about the study design can be found elsewhere [30,31,32]. In these analyses, we used baseline data collected at the screening visit, i.e., data collected before any intervention had been performed [32]. 

The sample size calculation in the Palliative-D study was based on the primary endpoint in the original study, i.e., the effect of vitamin D on pain, assessed with daily opioid use [30]. No separate power calculation was performed for this study. 

### 2.2. Ethics Statements

The study was approved by the Regional Ethical Committee in Stockholm (Dnr. 2017/405-31/1) and was conducted according to the declaration of Helsinki. All study participants received both oral and written information about the study and gave their written informed consent before any study related procedure was performed. Trial registration: Clinicaltrial.gov Identifier: NCT03038516; 31 January 2017.

### 2.3. Patient Demographics

All data was retrieved from the Palliative-D study eCRF database, stored at Karolinska Institutet. Data was anonymized before analysis and included information on sex, age, date of birth, cancer type and ongoing cancer treatment at the time of inclusion in the study. The study participants were categorized into four groups depending on their potential cancer-related eating difficulties/nutritional problems: gastrointestinal, gynecological, head–neck and other cancers. “Other” included e.g., prostate, lung and breast cancers. At the screening visit, blood samples were collected for the analysis of albumin and CRP levels. The patients also filled in the Edmonton Symptom Assessment System (ESAS) questionnaire [26], see details below. Based on data from Statistics Sweden, we assessed socioeconomic status using the average income of the patients’ area of residence as a proxy. The socioeconomic status was divided in two categories: living in an area above or below the average the national income. 

### 2.4. Biochemical Markers

Albumin (g/L) and CRP (mg/L) were analyzed by Karolinska University Laboratory at Karolinska University Hospital, Sweden using ISO 15189:2012 accredited methods. 

### 2.5. The Edmonton Symptom Assessment System

The Edmonton Symptom Assessment System (ESAS) is a validated assessment instrument used in clinical settings, as well as for research purposes, aimed at collecting disease-related self-reported symptoms, including appetite and fatigue, from individuals with advanced diseases [26]. ESAS measures the experience of symptoms over the last 24 h. 

Respondents are asked to rate nine core symptoms: pain, fatigue, nausea, depression, anxiety, tiredness, appetite, feeling of wellbeing and shortness of breath, on a numeric rating scale between 0 to 10, where 0 equals ‘no suffering’, and 10 ‘unbearable suffering’. Hence, a low rating equals less symptoms, and a high rating equals more symptoms. An optional tenth variable, QoL, was included in the Swedish version of ESAS [26], which was used in this study. 

The instrument was developed for use in palliative care in the 1990s and has been thoroughly validated and translated into more than 20 languages. It is used extensively in palliative care in both oncological and non-oncological settings [26,33,34,35]. Although no clear cut-off values have been determined [36], it has been suggested that ≥1 point in improvement and ≤1 point in deterioration for each of the included symptoms should be seen as a clinically significant change [37].

### 2.6. Primary and Secondary Outcomes

Appetite was analyzed as our exposure. Albumin was our primary outcome, and CRP, QoL, fatigue and pain were considered secondary outcomes. Appetite, QoL, fatigue and pain were all assessed with ESAS. There was no missing data except for one participant who had not answered the QoL question. 

### 2.7. Statistical Analysis

Descriptive statistics are presented as mean and standard deviation (SD), or number (*n*) and percentage (%). Differences between men and women were assessed using independent *t*-tests for continuous variables and chi-squared tests for categorical variables, respectively. 

Spearman’s rank test was used to assess correlations between appetite and albumin, and appetite and CRP, QoL, fatigue, and pain. Linear regression models were fitted to examine the associations between appetite and outcomes, to allow for adjustment of possible confounding factors including age, cancer type, cancer treatment and socioeconomic status. *p*-values < 0.05 were considered statistically significant. Statistical analyses were performed using Stata 16.1 (Stata Corporation, College Station, TX, USA). 

## 3. Results

### 3.1. Study Population

There were 266 men (mean age: 70 years) and 264 women (mean age: 67 years) included in the study. The mean survival time after baseline assessment was 235 days. The most common cancer type was gastrointestinal cancer (*n* = 218), including cancer in the upper and lower gastrointestinal tract, pancreas, bile ducts, liver and the esophagus. There was a statistically significant difference in age between men and women (*p* = 0.02). We found no differences between women and men in socioeconomic status, mean survival time measured in number of days, levels of albumin or CRP, or in the ESAS-variables of appetite, QoL, fatigue and pain. Patient characteristics are presented in Table 1.

### 3.2. Primary Outcome: Appetite and Albumin

**Figure 1:**
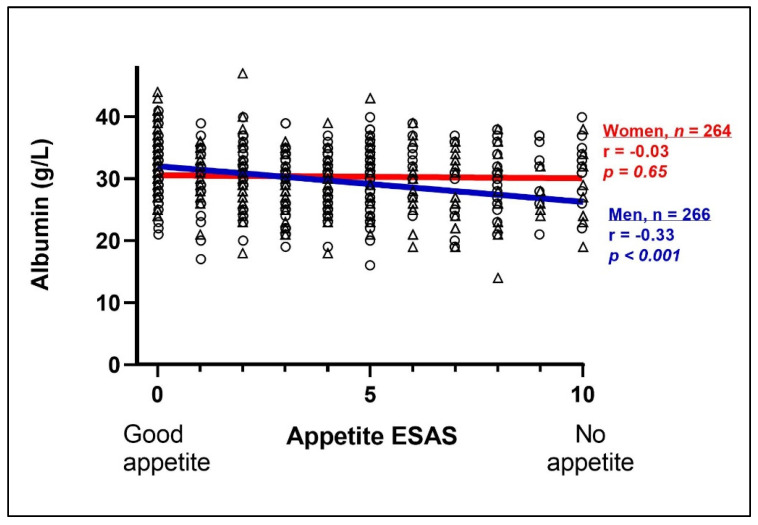
The relationship between appetite, assessed with Edmonton Symptom Assessment System (ESAS), and albumin (*n* = 530) in cancer patients in palliative care. Comparison between men (triangles, blue line) and women (circles, red line). Spearman’s correlation was used to calculate r- and *p*-values. ESAS score: 0 = good appetite, 10 = no appetite.

We found a correlation between appetite and albumin in men. A low appetite (i.e., a high ESAS score) was correlated with low albumin levels in men (r = −0.33, *p* ≤ 0.001), but not in women (r = −0.03, *p* = 0.65) (Figure 1). In the regression analysis, low appetite was significantly associated with low albumin in men β –0.57 (95% CI: −0.79, −0.36), but not in women, after adjustments for age, cancer type, cancer treatment and socioeconomic status (Table 2). 

### 3.3. Secondary Outcomes: Appetite and CRP, QoL, Fatigue and Pain

**Figure 2:**
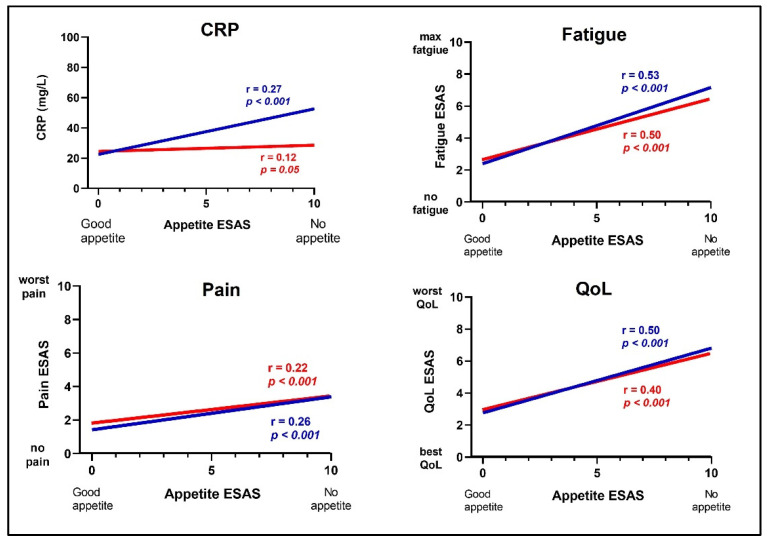
The relationship between appetite and CRP (*n* = 530), Quality of life (*n* = 529), fatigue (*n* = 530) and pain (*n* = 530), assessed using ESAS in cancer patients in palliative care; comparison between men (*n* = 266, blue lines) and women (*n* = 264, red lines). Spearman’s correlation was used to calculate r- and *p*-values. ESAS = Edmonton Symptom Assessment System. 0 = no suffering, 10 = unbearable suffering; CRP = C-Reactive protein; QoL = Quality of Life.

Lower appetite was significantly correlated with higher CRP (r = −0.27, *p* ≤ 0.001) in men, but not in women (r = −0.12, *p* = 0.05) (Figure 2). Appetite was significantly correlated with QoL, fatigue and pain (*p* for all ≤0.001) (Figure 2), in both men and women. Correspondingly, linear regression analysis showed a significant association between appetite and CRP in men but not in women, and a significant association between appetite and QoL, fatigue and pain in both sexes (Table 2). The results remained largely unchanged after adjustments for age, type of cancer, cancer treatment and socioeconomic status. 

## 4. Discussion

In this study, we showed that associations between appetite and albumin, and appetite and CRP, differ between men and women. We found that low appetite was significantly correlated with low albumin levels and high CRP levels only in men. Low appetite was correlated with impaired QoL and high fatigue and pain scores in both sexes. Thus, the results presented here highlight the impact of appetite in cancer patients, both as a prognostic marker and as a symptom, and show that it has an influence on QoL.

It is well known that cancer cachexia is associated with hypoalbuminemia and anorexia, i.e., loss of appetite [38,39]. However, to our knowledge, there are no previous studies on the association between albumin level and appetite in palliative care cancer patients; neither has anyone studied whether there is a difference between men and women in palliative care. Where albumin level is used as a prognostic marker, it may have a different impact in men and women. For example, in a large study from Austria (*n* = 285,930), low levels of albumin were associated with increased mortality in men, but not in women [40].

Our results are in line with a previous study showing an association between low appetite, high CRP levels and severe fatigue [20]. In another study, comprising 772 Portuguese cancer patients, it was reported that the impact of appetite on QoL was more pronounced in women than in men [41]. In our study, we confirm that there is a correlation between appetite and QoL, although we found an association in both sexes. However, our study was performed exclusively in palliative care patients, while the Portuguese study included oncological patients at various cancer stages. In line with our results, a large study on cancer patients in the USA (*n* = 954), also showed a significant correlation between appetite and QoL [38]. Other studies have shown associations between loss of appetite, malnutrition and impaired QoL [1,2]. However, analyses were not performed in men and women separately in any of these studies.

Inflammation leads to decreased albumin levels, elevated CRP and more intense pain, and is also thought to be an important factor in fatigue (19). Moreover, inflammation may lead to decreased appetite [40,42]. Thus, inflammation induced by cancer may explain the observed associations between appetite and albumin, CRP, fatigue, and pain. However, the association between low albumin and high CRP, i.e., inflammation, was only present in men, and not in women. Potentially, inflammation affects appetite more in men than in women, as it has been suggested that there is a sex-difference in the physiology of eating due to hormonal differences [43]. Nonetheless, it has not been reported that there are any differences based on sex in terms of appetite during starvation, where appetite is preserved, and cancer cachexia, where appetite is lost [44]. 

Interventions to stimulate appetite in patients in palliative care include pharmacological treatments using mirtazapine or short-term corticosteroids [6,9]. The anti-inflammatory effect of corticosteroids probably contributes to the appetite-stimulating effect, and it also has positive effects on both pain and fatigue—symptoms that we have shown to be associated with appetite. Corticosteroids are generally efficient in increasing appetite in both men and women, and seem to have no difference in effect between the sexes. 

This study has several limitations. Socioeconomic factors have previously been shown to affect appetite, where low socioeconomic status has been associated with higher energy intake and thereby also weight gain [45]. Further, Cheon et al. suggest that along with physiological and psychological influences, additional factors, such as subjective feelings of deprivation of social standing, also affect appetite [45]. The lack of information on socioeconomic factors such as educational level is, therefore, a limitation. Nevertheless, we were able to use a proxy for socioeconomic status based on the residential area of each participant having above or below average national income levels.

Patients diagnosed with gastrointestinal and head–neck cancers have a high risk of loss of appetite and/or eating difficulties [46,47], while patients with gynecological cancers involving peritoneal carcinosis have a high risk of ileus/subileus, which affects nutritional intake and appetite [48,49]. Ongoing oncological treatment may also affect not only appetite, but also albumin, CRP, fatigue, pain and QoL. Although our results remained similar after adjustment for possible confounding factors, it is a strength of our study that we were able to include cancer type, cancer treatment and socioeconomic status as potential confounding factors.

A major limitation is that we did not have data on the patients’ weight and body mass index. However, weight is a poor marker for assessment of sufficient energy intake in cancer patients since it is highly affected by metabolic derangements due to the cancer disease [50,51]. 

Another limitation is that we did not know the date of the patients’ cancer diagnosis. The time from diagnosis could span from weeks to several years before admission to palliative care. The time the patient had lived with the cancer disease may have affected their appetite, QoL, fatigue and pain scores. Further, there was a risk of selection bias. Only patients that had agreed to be screened for participation in a randomized, controlled trial on vitamin D treatment were included. Hence, patients that were reluctant to participate in the study for any reason, or too weak to be able to participate, were never included. In future studies, broader inclusion criteria would be preferable to strengthen the external validity of the results. Finally, this was a cross sectional study and so a causal relationship between the variables could not be investigated. Nevertheless, we believe that the results are still of value in clinical practice. 

A strength of the study was that this was a multicenter study, recruiting study participants from a broad geographic area. Palliative care patients were recruited regardless of age, cancer type, ongoing palliative oncological treatment and performance status. Thus, the participants were very heterogenous, which might be a limitation, but conversely, this might also be a strength, since it made the results more generalizable in the palliative care setting. 

Another strength was that we used the validated ESAS questionnaire [26] to assess appetite, fatigue and pain. However, a limitation was that the optional ESAS QoL variable used in this study has not been validated. ESAS is a well-established tool for symptom assessment in palliative care, and has been widely used for more than 30 years [26,33,34,35]. However, it only measures the symptoms over the last 24 h. In palliative cancer patients ESAS total scores might be less valid, as individual items might increase disproportionally as death approaches [16]. For that reason, we assessed the different symptoms independently, rather than using the total ESAS scores. Still, the questionnaire is a subjective measurement and is often more appropriate for use in studying intra-individual changes over time rather than comparisons between individuals in a cross-sectional manner. Nonetheless, we also had objective measurements of outcomes, such as albumin and CRP levels. 

## 5. Conclusions

In conclusion, our results indicated that the associations between appetite and the biochemical markers albumin and CRP in palliative care patients differ between men and women. We found that low albumin, and a high CRP, are associated with a low appetite in men, but not in women. The finding that appetite changes seem to affect albumin levels to a larger extent in men than in women adds new valuable knowledge for clinical practice in palliative care, since albumin is often used for assessing prognosis. Whether there is a sex difference in albumin as a prognostic marker needs to be explored in future studies. Moreover, our results reveal that appetite is associated with QoL, fatigue and pain in both sexes. The results bring a new perspective to the understanding of appetite that could be of value in clinical cancer care, and highlight the importance of assessing appetite in palliative care patients. 

## Figures and Tables

**Table 1:** Patient Demographics.

Variable	All (*n* = 530) Mean ± SD	Men (*n* = 266) Mean ± SD	Women (*n* = 264) Mean ± SD
Age, years	68.6 ± 11.2	69.8 ± 10.6	67.5 ± 11.7
Survival, days	235 ± 255	220 ± 211	248 ± 238
ESAS Appetite	3.5 ± 2.9	3.4 ± 2.9	3.6 ± 3.0
ESAS QoL	4.9 ± 2.5	4.2 ± 2.4	4.2 ± 2.6
ESAS Fatigue	4.0 ± 2.7	4.0 ± 2.7	4.0 ± 2.6
ESAS Pain	2.2 ± 2.3	2.1 ± 2.3	2.3 ± 2.4
Albumin (g/L)	30.3 ± 5.2	30.1 ± 5.3	30.5 ± 5.1
CRP (mg/L)	29.3 ± 46.4	32.7 ± 49.2	26.0 ± 43.1
	***n* (%)**	***n* (%)**	***n* (%)**
High socioeconomic status	251 (47.4)	119 (44.7)	132 (50.0)
**Cancer type**			
Gastrointestinal	218 (41.1)	126 (47.4)	92 (34.9)
Gynecological	39 (7.4)	0.00	39 (14.8)
Head and neck	11 (2.1)	9 (3.4)	2 (0.8)
Other	262 (49.4)	131 (49.3)	13 (49.6)
**Cancer treatment**			
Target therapy	38 (7.2)	20 (7.5)	18 (6.8)
Chemotherapy	276 (52.1)	126 (47.4)	150 (56.8)
No active treatment	151 (28.79	79 (29.7)	72 (27.7)
Hormones	55 (10.4)	36 (13.5)	19 (7.2)
Radiotherapy	9 (1.7)	5 (1.9)	4 (1.5)

ESAS = Edmont Symptom Assessment System. QoL = Quality of Life, CRP = C-reactive protein; High socioeconomic status = living in an area > average Swedish income.

**Table 2:** Linear regression models illustrating associations between self-reported appetite and quality of life (QoL), fatigue, pain, albumin and C-reactive protein (CRP) in palliative cancer patients.

	Crude Β (95% CI)	Adjusted for Age, Cancer Type, Cancer Treatment and SES
**All**		
Albumin (g/L)	−0.30 (−0.45, −0.15)	−0.30 (−0.45, −0.16)
CRP (mg/L)	1.63 (0.29, 3.0)	1.55 (0.20, 1.90)
ESAS QoL	0.38 (0.31, 0.44)	0.31 (0.31, 0.44)
ESAS Fatigue	0.43 (0.36, 0.50)	0.42 (0.35, 0.49)
ESAS Pain	0.18 (0.11, 0.25)	0.18 (0.11, 0.24)
**Men**		
Albumin (g/L)	−0.58 (−0.79, −0.37)	−0.57 (−0.79, −0.136)
CRP (mg/L)	3.0 (1.0, 5.0)	2.90 (0.86, 4.90)
ESAS QoL	0.41 (0.32, 0.50)	0.39 (0.30, 0.47)
ESAS Fatigue	0.48 (0.38, 0.57)	0.45 (0.35, 0.55)
ESAS Pain	0.20 (0.10, 0.30)	0.18 (0.08, 0.30)
**Women**		
Albumin (g/L)	−0.04 (−0.25, 0.17)	−0.06 (−0.27, 0.15)
CRP (mg/L)	0.42 (−0.35, 2.19)	0.52 (−1.26, 2.31)
ESAS QoL	0.53 (0.25, 0.55)	0.35 (0.25, 0.45)
ESAS Fatigue	0.38 (0.28, 0.48)	0.38 (0.30, 0.47)
ESAS Pain	0.16 (0.06, 0.26)	0.57 (0.79, 0.36)

SES = Socioeconomic status: Living in an area > average Swedish income or not. ESAS = Edmonton Symptom Assessment System. 0 = no suffering, 10 = unbearable suffering.

## Data Availability

Raw data from the “Palliative-D” study is available from the corresponding author upon request.
